# Acute Pancreatitis Associated with Atypical Bacterial Pneumonia: Systematic Literature Review

**DOI:** 10.3390/jcm11237248

**Published:** 2022-12-06

**Authors:** Gwendolyn Graf, Giulia A. M. Vassalli, Lisa Kottanattu, Mario G. Bianchetti, Carlo Agostoni, Gregorio P. Milani, Sebastiano A. G. Lava, Pietro B. Faré, Simone Janett

**Affiliations:** 1 Pediatric Institute of Southern Switzerland, Ente Ospedaliero Cantonale, 6500 Bellinzona, Switzerland; 2 Faculty of Biomedical Sciences, Università della Svizzera Italiana, 6900 Lugano, Switzerland; 3 Family Medicine, Faculty of Biomedical Sciences, Università della Svizzera Italiana, 6900 Lugano, Switzerland; 4 Pediatric Unit, Fondazione IRCCS Ca’ Granda Ospedale Maggiore Policlinico, 20122 Milan, Italy; 5 Department of Clinical Sciences and Community Health, Università degli Studi di Milano, 20124 Milan, Italy; 6 Pediatric Cardiology Unit, Department of Pediatrics, Centre Hospitalier Universitaire Vaudois, University of Lausanne, 1011 Lausanne, Switzerland; 7 Heart Failure and Transplantation, Department of Paediatric Cardiology, Great Ormond Street Hospital, London WC1N 1DZ, UK; 8 Division of Infectious Diseases, Department of Medicine, Ente Ospedaliero Cantonale, 6900 Lugano, Switzerland; 9 Department of Pulmonology, University Hospital Zurich, 8091 Zurich, Switzerland

**Keywords:** atypical pneumonia, acute pancreatitis, *Coxiella burnetii*, *Legionella* species, *Mycoplasma pneumoniae*

## Abstract

Background: Extra-pulmonary features sometimes occur in association with atypical bacterial pneumonia and include neurologic manifestations, diarrhea, rashes, altered liver enzymes, or kidney injury, among other conditions. Acute pancreatitis has been associated with atypical pneumonias since 1973. Methods: We performed a systematic review of the literature in the Excerpta Medica, National Library of Medicine, and Web of Science databases. We retained 27 reports published between 1973 and 2022 describing subjects with an otherwise unexplained pancreatitis temporally associated with an atypical pneumonia. Results: The reports included 33 subjects (19 males, and 14 females; 8 children and 25 adults) with acute pancreatitis temporally associated with atypical pneumonia caused by *Mycoplasma pneumoniae* (*n* = 18), *Legionella* species (*n* = 14), or *Coxiella burnetii* (*n* = 1). Approximately 90% of patients (*n* = 29) concurrently presented with respiratory and pancreatic diseases. No cases associated with *Chlamydophila pneumoniae*, *Chlamydophila psittaci*, or *Francisella* species were found. Conclusions: Acute pancreatitis has been associated with various infectious agents. The present review documents the association with atypical pneumonia induced by *Mycoplasma pneumoniae*, *Legionella* species, and *Coxiella burnetii*.

## 1. Introduction

The term atypical bacterial pneumonia denotes pulmonary infections caused by *Chlamydophila pneumoniae*, *Chlamydophila psittaci*, *Coxiella burnetii*, *Francisella* species, and especially *Legionella* species and *Mycoplasma pneumoniae* [1,2]. These bacteria are responsible for 15–20% of community-acquired pneumonia cases [1,2].

Non-pulmonary features are common in patients affected by atypical bacterial pneumonia conditions [2,3,4,5]. The reported non-pulmonary features include abdominal pain, nausea or vomiting, ileus, diarrhea, jaundice, elevated aminotransferases, nervous system dysfunction (including headache, mental confusion, and reduced levels of consciousness), acute kidney injury, and skin rashes, among others. Interestingly, the non-pulmonary features of patients with an atypical bacterial pneumonia syndrome may precede the respiratory disease, may present concomitantly with the respiratory disease, or after the respiratory disease [2,3,4,5].

Acute pancreatitis has been associated with atypical bacterial pneumonia since 1973 [6]. As this issue has never been systematically evaluated, we performed a systematic review of the literature. The study aimed to gain insight into the features of pancreatitis associated with atypical pneumonias and to speculate on the mechanisms underlying acute pancreatitis in these patients.

## 2. Materials and Methods

### 2.1. Search Strategy

We performed a structured literature search with no date or language restrictions in the databases Excerpta Medica, National Library of Medicine, and Web of Science, in accordance with the guidelines of the 2020 version of the Preferred Reporting of Systematic Reviews and Meta-Analyses [7]. Search terms were (“atypical pneumonia” OR “*Chlamydia pneumoniae*” OR “*Chlamydia psittaci*” OR “*Chlamydophila pneumoniae*” OR “*Chlamydophila psittaci*” OR “*Coxiella burnetii*” OR “*Francisella tularensis*” OR “*Legionella*” OR “*Mycoplasma pneumoniae*”) AND (“acute pancreatitis”). The bibliography of each identified report was also screened for secondary references. Additionally, to detect as many cases as possible, articles published in non-indexed journals were also evaluated. The search was carried out in April 2022 and was repeated before submission.

### 2.2. Eligibility Criteria—Case Selection

All original articles or letters reporting humans with a community-acquired pneumonia caused by *Chlamydophila (Chlamydia) pneumoniae*, *Chlamydophila (Chlamydia) psittaci*, *Coxiella burnetii*, *Francisella tularensis*, *Legionella* species, or *Mycoplasma pneumoniae* temporally associated with an acute pancreatitis were considered eligible. Inclusion was restricted to apparently immunocompetent subjects. The diagnosis of pneumonia caused by an atypical pathogen was only retained in cases with both a characteristic clinical presentation and appropriate microbiology laboratory testing [1,2]. A diagnosis of acute pancreatitis was made in patients with an increase in amylase or lipase values to >3 times the upper normal laboratory limit, irrespective of the clinical and imaging features [8]. The Institutional Review Board authorization was not a prerequisite for this systematic literature review. After an initial selection round based on the title and abstract, the eligibility of the full text of the selected reports was assessed. The gray literature was assessed in the same way.

### 2.3. Data Extraction

The following four groups of data were collected from each patient, using a predesigned data extraction form: (1) demographics, pre-existing conditions—with emphasis on recognized precipitants of acute pancreatitis (chronic alcohol use disorder, gallstones, hypercalcemia, increased triglyceride levels, and medication with drugs [9] implicated as a causative agent for acute pancreatitis), and microbiology laboratory testing; (2) the temporal relationship between respiratory disease and the onset of pancreatitis (the term pre-infectious denoted cases with pancreatitis preceding pneumonia by ≤10 days, the term intra-infectious denoted cases with concomitant presentation of pneumonia and pancreatitis, and the term post-infectious denoted cases with pneumonia preceding pancreatitis by ≤10 days), abdominal involvement (pain, nausea or vomiting, ileus, diarrhea, and jaundice), an increase in alanine or aspartate aminotransferase levels (more than twice the upper limit of normal), and imaging studies to categorize pancreatitis as interstitial edematous or necrotizing [9,10]; (3) non-pulmonary features including central nervous system dysfunction (headache, mental confusion, and reduced levels of consciousness), the occurrence of acute kidney injury using the KDIGO criteria [11], the occurrence of multiple organ dysfunction [12], and skin rashes; (4) the length of hospitalization and the occurrence of death.

The literature search, the selection of reports retained for analysis, and the data extraction process were independently carried out by two authors in an unblinded fashion with the support of an experienced investigator. Two authors entered the data into a pilot-tested database, and the experienced investigator verified the accuracy of the data entries.

### 2.4. Comprehensiveness of Reporting—Analysis

Each of the four groups of extracted data was rated for completeness [13] (0, 1, or 2) and the reporting quality was graded according to the sum (excellent (≥6), good (4–5), or acceptable (3–4)).

Pairwise deletion was used to deal with any missing data. The categorical data are shown as counts and were analyzed using Fisher’s exact test. The continuous data are presented as medians and interquartile ranges and were analyzed using the Mann–Whitney–Wilcoxon U test. Two-sided *p* values of <0.05 were considered to be significant.

## 3. Results

**Figure 1:**
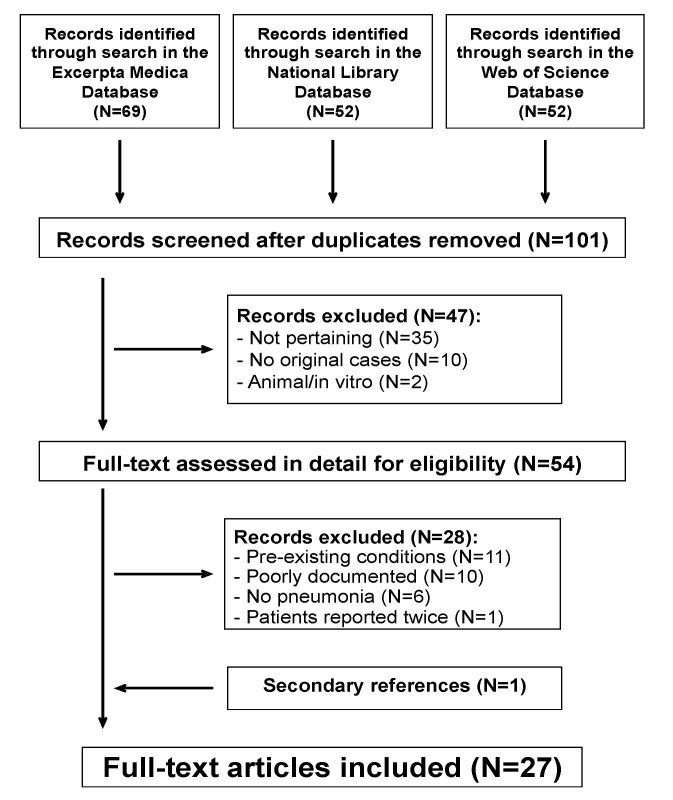
Acute pancreatitis associated with atypical bacterial pneumonia. Flowchart of the literature search.

The literature search process is outlined in Figure 1.

Six reports were excluded because lacking clinical signs and symptoms of pneumonia. Swedish patients reported twice in the literature [6,14] were considered only once. For the final analysis, we retained 27 reports [6,14,15,16,17,18,19,20,21,22,23,24,25,26,27,28,29,30,31,32,33,34,35,36,37,38,39] published between 1973 and 2022 from France (*n* = 7), the United States of America (*n* = 3), Belgium (*n* = 3), Sweden (*n* = 3), Spain (*n* = 2), Denmark (*n* = 1), the United Kingdom (*n* = 1), Germany (*n* = 1), Italy (*n* = 1), Japan (*n* = 1), India (*n* = 1), South Korea (*n* = 1), Switzerland (*n* = 1), and Venezuela (*n* = 1). Eighteen articles were written in English, four in French, three in Spanish, one in German, and one in Danish.

The reports included 33 subjects with pancreatitis temporally associated with an atypical pneumonia: 18 cases associated with *Mycoplasma pneumoniae* [6,14,15,16,17,18,19,20,21,22,23,24,25,26], 14 cases associated with *Legionella* species [27,28,29,30,31,32,33,34,35,36,37,38], and 1 case associated with *Coxiella burnetii* [39]. The reports did not include any case of acute pancreatitis temporally associated with pneumonia caused by *Chlamydophila pneumoniae*, *Chlamydophila psittaci*, or *Francisella* species.

The completeness of reporting was excellent in 19 cases (*Legionella*, *n* = 11; *Mycoplasma*, *n* = 8), good in 8 cases (*Mycoplasma*, *n* = 5; *Legionella*, *n* = 3), and acceptable in the remaining 6 cases (*Mycoplasma*, *n* = 5; *Coxiella*, *n* = 1).

### 3.1. Microbiological Diagnosis

The microbiological diagnosis of *Mycoplasma pneumoniae* infection was made by detecting a significant rise in immunoglobulin G titer levels when comparing acute and convalescent blood samples (*n* = 17) or both an antibody titer and a positive *Mycoplasma* test in a respiratory tract sample (*n* = 1). The laboratory diagnosis of *Legionella* infection was made by means of a positive sputum or tissue testing (*n* = 6), a rise in immunoglobulin G titer levels (*n* = 5), or a positive urinary test (*n* = 3). The diagnosis of *Coxiella* infection was made by means of a rise in immunoglobulin G titer levels (*n* = 1).

### 3.2. Clinical and Laboratory Features

The characteristics of the 32 patients are presented in Table 1. Patients with atypical pneumonia and acute pancreatitis associated with *Mycoplasma pneumoniae* were more frequently male (*p* = 0.0116) and younger (*p* = 0.0112) than those with *Legionella* species infection. Approximately 90% of patients presented with concurrent respiratory and pancreatic disease. None of the 33 patients were affected by chronic alcohol use disorder, hypercalcemia, and hypertriglyceridemia, or underwent treatment with a drug potentially associated with acute pancreatitis. A 62-year-old man concurrently presented with an asymptomatic cholelithiasis [14].

The following abdominal features were observed: abdominal pain, nausea or vomiting, ileus, diarrhea, jaundice, and elevated aminotransferases. Diarrhea (*p* = 0.0099), jaundice (*p* = 0.0278), and elevated liver enzymes (*p* = 0.0002) were more commonly observed in cases caused by *Legionella*.

No pancreatic imaging studies were carried out in 12 cases. The following imaging studies were performed in the remaining patients: ultrasound and computed tomography (*n* = 9); ultrasound (*n* = 8); and computed tomography (*n* = 4). Edema was identified following imaging in 14 cases and necrosis in 5 cases, respectively.

The prevalence of central nervous system dysfunction (*p* = 0.0002) and acute kidney injury (*p* = 0.0037) was higher in *Legionella* cases than in *Mycoplasma* cases. A skin rash was rarely reported.

Multi-organ dysfunction occurred in seven cases. Two of them, a 68-year-old female reported in 1974 [14] and a 66-year-old man reported in 1986 [29], died.

## 4. Discussion

Acute pancreatitis mostly occurs in subjects with chronic alcohol use disorders, cholelithiasis or choledocholithiasis, hypercalcemia, and hypertriglyceridemia, or is drug-induced [8,9]. The present systematic review demonstrates that acute pancreatitis may be temporally associated with a community-acquired atypical pneumonia syndrome caused by *Mycoplasma pneumoniae, Legionella* species, or *Coxiella burnetii* (both in childhood and adulthood). In contrast, no cases associated with the remaining bacterial pathogens of atypical pneumonia were found.

The pathophysiology of acute pancreatitis associated with atypical pneumonia caused by *Mycoplasma pneumoniae* or *Legionella* species is elusive. *Mycoplasma pneumonia* is occasionally followed by an acute glomerulonephritis histologically characterized by distinctive immune deposits [4,5,40]. Both *Legionella* and *Mycoplasma* may present concomitantly with respiratory disease and acute kidney injury, which is not immune-mediated, but directly caused by the pathogen or by the release of pro-inflammatory mediators [41]. In this study, pneumonia and pancreatitis occurred concurrently in most cases, suggesting that pancreatitis is not immune-mediated, and is directly caused by the pathogen or by the release of pro-inflammatory mediators [42]. 

Interestingly, pro-inflammatory cytokines, such as interleukin-1β, interleukin-6, and tumor necrosis factor-α, have been implicated in both atypical pneumonia [41] and in acute pancreatitis [42], including the experimental pancreatitis induced by the administration of cerulein (a cholecystokinin analog), among others [43,44].

Immunoglobulin M antibodies against *Mycoplasma pneumoniae* are detected [45] in blood in approximately one-third of patients with acute pancreatitis (and some patients with acute meningoencephalitis). It has therefore been postulated that, during pancreatitis, antigenic sequences similar to the major antigens of *Mycoplasma pneumoniae* are revealed, which elicit an immunoglobulin M antibody response [45]. For this reason [46], only acute pancreatitis cases associated both with acute respiratory disease and an appropriate microbiology laboratory test were included in the present analysis (an immunoglobulin M detection was not accepted as a stand-alone diagnostic test).

The practical relevance of this literature review is three-fold. First, acute pancreatitis can be added to the list of possible causes of abdominal symptoms in patients with atypical bacterial pneumonia. Patients with pancreatitis typically complain of upper abdominal and back pain, often associated with nausea and vomiting. Second, the diagnosis of atypical pneumonia deserves consideration in pancreatitis cases without a precipitant (such as gallstones, alcohol use disorders, hypercalcemia, and hypertriglyceridemia) or without any medication implicated as causative for acute pancreatitis. Finally, pancreatitis adds to the already rather long list of non-pulmonary features associated with atypical bacterial pneumonia.

First-line treatment options which are currently recommended for *Mycoplasma* infections include the macrolide azithromycin, the tetracycline doxycycline (doxycycline is unlikely to cause tooth discoloration in young children, contrary to other tetracyclines), or a respiratory fluoroquinolone (i.e., levofloxacin or moxifloxacin) [1]. Azithromycin or levofloxacin are the preferred antimicrobials for *Legionella* infections [1]. Acute *Coxiella* infections are treated with doxycycline [1]. No cases of pancreatitis have been causally associated with azithromycin, doxycycline, levofloxacin, or moxifloxacin [9].

The results of this literature review must be viewed with an understanding of the inherent limitations of the analysis, which included data from less than 30 reports published over a period of approximately 50 years. The available information did not allow the severity of pancreatitis to be stratified using a recognized classification. Moreover, a temporal association between an atypical bacterial pneumonia and pancreatitis does not necessarily imply causality. The prevalence of pancreatitis complicating atypical bacterial pneumonia is currently unknown (but is likely low). Finally, the analysis did not address atypical pneumonias triggered by viral pathogens, including respiratory syncytial viruses, parainfluenza viruses, influenza viruses, paramyxoviruses, and adenoviruses, among others. Recent data suggest that severe acute respiratory syndrome coronavirus 2 may also be associated with acute pancreatitis [47].

## 5. Conclusions

Acute pancreatitis has been associated with infectious agents such as mumps virus, coxsackieviruses, cytomegalovirus, hepatitis B virus, herpes simplex virus, severe acute respiratory syndrome coronavirus 2, varicella-zoster virus, aspergillus, ascaris, cryptosporidium, toxoplasma, leptospira, and salmonella [48,49]. The present review of the literature documents the association with *Mycoplasma pneumoniae*, *Legionella* species, and *Coxiella burnetii*. 

## Figures and Tables

**Table 1:** Characteristics of 33 patients (3 to 88 years of age with atypical bacterial pneumonia associated with acute pancreatitis). Data are presented as the frequency (with percentage) or median (with interquartile range).

	All Cases	*Mycoplasma* *pneumoniae*	*Legionella* *Species*	*Coxiella* *burnetii*	*p*-Values ^⤬^
***n***	33	18	14	1	
**Females:Males, *n***	14:19	11:7	2:12	1:0	**0.0116**
**Age**					
years	43 (21–56)	25 (13–52)	51 (45–57)	30	**0.0112**
<20 years, *n* (%)	8 (24)	8 (47)	0 (0)	0 (0)	**0.0044**
**Temporal relationship to pneumonia**					
Pre-infectious, *n* (%)	1 (3.0)	0 (0)	0 (0)	1 (0)	0.9999
Intra-infectious, *n* (%)	29 (88)	16 (89)	13 (93)	0 (0)	0.9999
Post-infectious, *n* (%)	3 (9.1)	2 (11)	1 (7.1)	0 (0)	0.9999
**Abdominal features**					
Abdominal pain, *n* (%)	25 (76)	16 (89)	9 (64)	0 (0)	0.1948
Nausea, vomiting, *n* (%)	14 (44)	10 (56)	4 (29)	0 (0)	0.7249
Ileus, *n* (%)	7 (21)	5 (28)	2 (14)	0 (0)	0.4264
Diarrhea, *n* (%)	5 (15)	0 (0)	5 (36)	0 (0)	**0.0099**
Jaundice, *n* (%)	4 (12)	0 (0)	4 (29)	0 (0)	**0.0278**
Elevated aminotransferases, *n* (%)	13 (39)	2 (11)	11 (79)	0 (0)	**0.0002**
**Pancreatic imaging**					
Interstitial edematous, *n* (%)	14 (42)	6 (33)	8 (57)	0 (0)	0.1570
Necrotizing, *n* (%)	5 (15)	3 (17)	2 (14)	0 (0)	0.9999
**Further features**					
Central nervous system dysfunction, *n* (%)	15 (45)	3 (17)	12 (86)	0 (0)	**0.0002**
Acute kidney injury, *n* (%)	9 (27)	1 (5.6)	8 (57)	0 (0)	**0.0037**
Multiple-organ dysfunction, *n* (%)	7 (21)	4 (22)	3 (21)	0 (0)	0.9999
Skin rashes, *n* (%)	1 (3.0)	1 (5.6)	0 (0)	0 (0)	0.9999
**Length of hospitalization, days**	25 (19–36)	21 (21–35)	29 (20–41)	14	0.4542
**Death, *n* (%)**	2 (6.1)	1 (5.6)	1 (7.1)	0 (0)	0.9999

**^⤬^***Mycoplasma pneumoniae* vs. *Legionella* species.

## Data Availability

Data are available upon reasonable request to the corresponding authors.

## References

[1] Basarab M., Macrae M.B., Curtis C.M. (2014). Atypical pneumonia. Curr. Opin. Pulm. Med..

[2] Cunha B.A., Ortega A.M. (1996). Atypical pneumonia. Extrapulmonary clues guide the way to diagnosis. Postgrad. Med..

[3] Terraneo L., Lava S.A.G., Camozzi P., Zgraggen L., Simonetti G.D., Bianchetti M.G., Milani G.P. (2015). Unusual eruptions associated with *Mycoplasma pneumoniae* respiratory infections: Review of the literature. Dermatology.

[4] Simoni C., Camozzi P., Faré P.B., Bianchetti M.G., Kottanattu L., Lava S.A.G., Milani G.P. (2020). Myositis and acute kidney injury in bacterial atypical pneumonia: Systematic literature review. J. Infect. Public Health.

[5] Betti C., Camozzi P., Gennaro V., Bianchetti M.G., Scoglio M., Simonetti G.D., Milani G.P., Lava S.A.G., Ferrarini A. (2021). Atypical bacterial pathogens and small-vessel leukocytoclastic vasculitis of the skin in children: Systematic literature review. Pathogens.

[6] Mårdh P.A., Ursing B. (1973). Acute pancreatitis in mycoplasma pneumoniae infections. Br. Med. J..

[7] Page M.J., McKenzie J.E., Bossuyt P.M., Boutron I., Hoffmann T.C., Mulrow C.D., Shamseer L., Tetzlaff J.M., Akl E.A., Brennan S.E. (2021). The PRISMA 2020 statement: An updated guideline for reporting systematic reviews. J. Clin. Epidemiol..

[8] Carroll J.K., Herrick B., Gipson T., Lee S.P. (2007). Acute pancreatitis: Diagnosis, prognosis, and treatment. Am. Fam. Physician.

[9] Badalov N., Baradarian R., Iswara K., Li J., Steinberg W., Tenner S. (2007). Drug-induced acute pancreatitis: An evidence-based review. Clin. Gastroenterol. Hepatol..

[10] Banks P.A., Bollen T.L., Dervenis C., Gooszen H.G., Johnson C.D., Sarr M.G., Tsiotos G.G., Vege S.S. (2013). Acute Pancreatitis Classification Working Group. Classification of acute pancreatitis-2012: Revision of the Atlanta classification and definitions by international consensus. Gut.

[11] Kellum J.A., Lameire N., Aspelin P., Barsoum R.S., Burdmann E.A., Goldstein S.L., Herzog C.A., Joannidis M., Kribben A., Levey A.S. (2012). Kidney Disease: Improving Global Outcomes (KDIGO) Acute Kidney Injury Work Group. KDIGO clinical practice guideline for acute kidney injury. Kidney Int. Suppl..

[12] Johnson D., Mayers I. (2001). Multiple organ dysfunction syndrome: A narrative review. Can. J. Anaesth..

[13] Murad M.H., Sultan S., Haffar S., Bazerbachi F. (2018). Methodological quality and synthesis of case series and case reports. BMJ Evid. Based Med..

[14] Mårdh P.A., Ursing B. (1974). The occurrence of acute pancreatitis in *Mycoplasma pneumoniae* infection. Scand. J. Infect. Dis..

[15] Schmid E., Blaich E. (1976). Akute Pankreatitis bei Infektion durch *Mycoplasma pneumoniae* (Acute pancreatitis in *Mycoplasma pneumoniae infections*). Z. Gastroenterol..

[16] Herbaut C., Tielemans C., Burette A., Dratwa M. (1983). *Mycoplasma pneumoniae* infection and acute pancreatitis. Acta Clin. Belg..

[17] Arriero Marín J.M., Gil Carbonell J., Mora Rufete A., Shum C. (1989). Pancreatitis y hepatitis en el curso de neumonía por *Mycoplasma pneumoniae* (Pancreatitis and hepatitis in pneumonia caused by *Mycoplasma pneumoniae*). Rev. Clin. Esp..

[18] Van Bever H.P., Van Doorn J.W., Demey H.E. (1992). Adult respiratory distress syndrome associated with *Mycoplasma pneumoniae* infection. Eur. J. Pediatr..

[19] Theissen O., Kempf J., Loeb J.P. (1994). Pancréatite aigue chez l’enfant, associée à un taux élevé d’anticorps anti-*Mycoplasma pneumoniae* (Acute pancreatitis in children, combined with high level of anti-*Mycoplasma pneumoniae* antibodies). Ann. Fr. Anesth. Reanim..

[20] Vic P., Blondin G., Blayo M., Finel E., Daaboul M., Queinnec C., Broussine L. (2004). Pancréatite aiguë et infection à *Mycoplasma pneumoniae* (Acute pancreatitis and *Mycoplasma pneumoniae* infection). Arch. Pédiatr..

[21] Nakagawa M., Ogino H., Shimohira M., Hara M., Shibamoto Y. (2009). Continuous regional arterial infusion therapy for acute necrotizing pancreatitis due to *Mycoplasma pneumoniae* infection in a child. Cardiovasc. Intervent. Radiol..

[22] Ficko C., Mellon G., Andriamanantena D., Merens A., Rapp C. (2011). Pancréatite aiguë à *Mycoplasma pneumoniae* (Acute *Mycoplasma pneumonia* pancreatitis). Med. Mal. Infect..

[23] Hopp E., Martínez L.C., Díaz M., Quintero A.V., Dominguez M., Di Girolamo C., Carreiro M. (2013). Neumonía por *Mycoplasma pneumoniae* complicada con pancreatitis y hepatitis aguda: A propósito de un caso (*Mycoplasma pneumoniae* pneumonia complicated with acute pancreatitis and hepatitis: A case study). Rev. Soc. Venez Gestroenterol..

[24] Yang A., Kang B., Choi S.Y., Cho J.B., Kim Y.J., Jeon T.Y., Choe Y.H. (2015). Acute necrotizing pancreatitis associated with *Mycoplasma pneumoniae* infection in a child. Pediatr. Gastroenterol. Hepatol. Nutr..

[25] Benzaquen M., Lebowitz D., Belenotti P., Durand J.M., Serratrice J. (2016). Acute pancreatitis and pneumonia due to *Mycoplasma pneumoniae*: A case report. BMC Res. Notes.

[26] Khan H.R.A., Singh A., Usman O., Rafiq S., Amin A. (2022). Acute Pancreatitis: An Unusual Extrapulmonary Manifestation of *Mycoplasma pneumoniae*. Cureus.

[27] Gordan V., Postic B., Zmyslinski R.W., Khan A.H. (1980). Legionnaires’ disease complicated by acute pancreatitis: Case report. Mil. Med..

[28] Jespersen C., Engbaek K. (1982). Legionaersygdom med pancreaspåvirkning (Legionnaires’ disease with pancreatic involvement). Ugeskr. Laeger.

[29] Michel O., Naeije N., Csoma M., Sergysels R., de Coster A. (1985). Acute pancreatitis in Legionnaires’ disease. Eur. J. Respir. Dis..

[30] Bollaert P.E., Maurizi M., Laprevote-Heully M.C., Lambert H., Larcan A. (1986). Pancréatite aiguë nécrotico-hémorrhagique au cours d’une maladie des légionnaires (Acute necrotic-hemorrhagic pancreatitis in Legionnaires’ disease). Presse Med..

[31] Eitrem R., Forsgren A., Nilsson C. (1987). Pneumonia and acute pancreatitis most probably caused by a *Legionella longbeachae* infection. Scand. J. Infect. Dis..

[32] Craven D.E., Mark E.J. (1987). Case records of the Massachusetts General Hospital. Weekly clinicopathological exercises. Case 37-1987. A 50-year-old man with bilateral pneumonia and respiratory failure. N. Engl. J. Med..

[33] Westblom T.U., Hamory B.H. (1988). Acute pancreatitis caused by *Legionella pneumophila*. South Med. J..

[34] Kesavan C.R., Pitchumoni C.S., Marino W.D. (1993). Acute painless pancreatitis as a rare complication in Legionnaires disease. Am. J. Gastroenterol..

[35] Mégarbane B., Montambault S., Chary I., Guibert M., Axler O., Brivet F.G. (2000). Acute pancreatitis caused by severe *Legionella pneumophila* infection. Infection.

[36] Hadef H., Bilbault P., Arzouq H., Berna C., Phelipot J.Y., Jaeger A. (2006). Violent abdominal pain: Severe *Legionella pneumophila* lung infection with acute pancreatitis. Am. J. Emerg. Med..

[37] Puerto Alonso J.L., Díaz de Souza P., Miragaya García D., Sánchez Porto A. (2011). Pancreatitis y colostasis disociada agudas: Manifestación inusual en la infección por *Legionella pneumophila* (Pancreatitis and dissociated cholestasis: An unusual manifestation in *Legionella pneumophila* induced-infections). Rev. Clin. Esp..

[38] Franchini S., Marinosci A., Ferrante L., Sabbadini M.G., Tresoldi M., Dagna L. (2015). Pancreatic involvement in *Legionella pneumonia*. Infection.

[39] Cancela Costa A., Chheang C., Thorens O., Lamy O., Prella M., Babaker M., Lamoth F., Greub G. (2021). Pancreatitis, hypereosinophilia and bilateral pulmonary infiltrates as presentation of acute Q fever. New Microbes New Infect..

[40] Poddighe D. (2018). Extra-pulmonary diseases related to *Mycoplasma pneumoniae* in children: Recent insights into the pathogenesis. Curr. Opin. Rheumatol..

[41] Brewster U.C. (2004). Acute renal failure associated with legionellosis. Ann. Intern. Med..

[42] Sah R.P., Garg P., Saluja A.K. (2012). Pathogenic mechanisms of acute pancreatitis. Curr. Opin. Gastroenterol..

[43] Esposito E., Campolo M., Casili G., Lanza M., Franco D., Filippone A., Peritore A.F., Cuzzocrea S. (2018). Protective effects of xyloglucan in association with the polysaccharide gelose in an experimental model of gastroenteritis and urinary tract Infections. Int. J. Mol. Sci..

[44] Cordaro M., Fusco R., D’Amico R., Siracusa R., Peritore A.F., Gugliandolo E., Genovese T., Crupi R., Mandalari G., Cuzzocrea S. (2020). Cashew (*Anacardium occidentale* L.) nuts modulate the Nrf2 and NLRP3 pathways in pancreas and lung after induction of acute pancreatitis by cerulein. Antioxidants.

[45] Leinikki P.O., Panzar P., Tykkä H. (1978). Immunoglobulin M antibody response against *Mycoplasma pneumoniae* lipid antigen in patients with acute pancreatitis. J. Clin. Microbiol..

[46] Landry M.L. (2016). Immunoglobulin M for acute infection: True or false?. Clin. Vaccine Immunol..

[47] Correia de Sá T., Soares C., Rocha M. (2021). Acute pancreatitis and COVID-19: A literature review. World J. Gastrointest. Surg..

[48] Parenti D.M., Steinberg W., Kang P. (1996). Infectious causes of acute pancreatitis. Pancreas.

[49] Rawla P., Bandaru S.S., Vellipuram A.R. (2017). Review of infectious etiology of acute pancreatitis. Gastroenterol. Res..

